# Adipose-Derived Mesenchymal Stem Cells in the Regeneration of Vocal Folds: A Study on a Chronic Vocal Fold Scar

**DOI:** 10.1155/2016/9010279

**Published:** 2016-01-06

**Authors:** Angelou Valerie, Kalodimou Vassiliki, Messini Irini, Psychalakis Nikolaos, Eleftheria Karampela, Papalois Apostolos

**Affiliations:** ^1^Phoniatrics and Applied Otorhinolaryngology Center, Amalias Avenue 42, 10542 Athens, Greece; ^2^Department of Flow Cytometry-Research & Regenerative Medicine, IASO Maternity Hospital, Kifissias Avenue 37-39, Maroussi, 15123 Athens, Greece; ^3^Pathology Department, IASO Maternity Hospital, Kifissias Avenue 37-39, Maroussi, 15123 Athens, Greece; ^4^Experimental & Research Center, ELPEN, 95 Marathonos Avenue, Pikermi, Attica, 190 09 Athens, Greece

## Abstract

*Background*. The aim of the study was to assess the histological effects of autologous infusion of adipose-derived stem cells (ADSC) on a chronic vocal fold scar in a rabbit model as compared to an untreated scar as well as in injection of hyaluronic acid.* Study Design*. Animal experiment.* Method*. We used 74 New Zealand rabbits. Sixteen of them were used as control/normal group. We created a bilateral vocal fold wound in the remaining 58 rabbits. After 18 months we separated our population into three groups. The first group served as control/scarred group. The second one was injected with hyaluronic acid in the vocal folds, and the third received an autologous adipose-derived stem cell infusion in the scarred vocal folds (ADSC group). We measured the variation of thickness of the lamina propria of the vocal folds and analyzed histopathologic changes in each group after three months.* Results*. The thickness of the lamina propria was significantly reduced in the group that received the ADSC injection, as compared to the normal/scarred group. The collagen deposition, the hyaluronic acid, the elastin levels, and the organization of elastic fibers tend to return to normal after the injection of ADSC.* Conclusions*. Autologous injection of adipose-derived stem cells on a vocal fold chronic scar enhanced the healing of the vocal folds and the reduction of the scar tissue, even when compared to other treatments.

## 1. Introduction

The human vocal fold consists of three layers: the epithelium, the lamina propria, and the vocal muscle. The epithelium of the vocal fold is a stratified squamous cell epithelium [1]. At the boundary between the epithelium and the underlying lamina propria there is a thin supportive structure formed from collagen, called basal lamina. The lamina propria in human vocal folds consists of three layers. The superficial layer of the lamina propria consists of myofibroblasts and macrophages, although the extracellular matrix is composed of collagen, elastin, hyaluronic acid, and fibronectin. The intermediate layer also has macrophages and myofibroblasts but the principal element of the extracellular matrix is the hyaluronic acid and the elastic fibers. The deep layer of the lamina propria is mostly composed of collagen, macrophages, and fibroblasts. This 3-layered structure and the specific microstructure provide the unique vibratory properties of the human vocal folds [2–5].

Vocal fold trauma due to inflammation, injury, radiotherapy, or surgery can lead to the formation of scar tissue. The microstructure of the lamina propria changes drastically by losing the distinctive 3-layer structure. There is an increase of collagen formation to the full depth of the lamina propria of scarred vocal folds which seems to form thick, organized bundles. The elastic fibers are present in the three layers but are disorganized and although the hyaluronic acid has the same density compared to the normal vocal fold, its distribution is uniform in all three layers of the lamina propria and even more accentuated in the deep layers [6–8]. This scar tissue resulting from the healing process causes stiffness and modifies the unique biomechanical properties of the vocal fold. The mucosal wave during phonation is altered and the patient presents with dysphonia.

Numerous phonosurgical procedures have been attempted to restore the vibratory properties of the scarred vocal folds. This includes injection of collagen [9], hyaluronic acid [10, 11], hydroxyapatite [12], acellular xenogenic matrix [13], autologous fat [14], or even thyroplasty type III and scar excision. All these surgical treatments are not exempt from complications such as formation of granulation or stiffness of the remaining vocal fold. Voice therapy is a more conservative treatment with various results. None of them had been attempted with an optimal result.

**Scheme 1:**
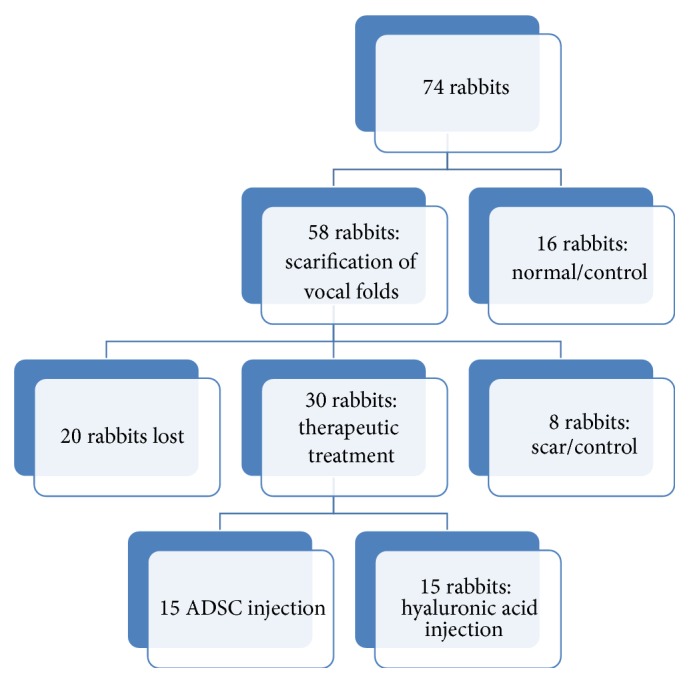
Plan of protocol.

Recently, several studies have been performed in animal models regarding the effect of cell therapy on scarred vocal folds, human embryonic stem cells [15], human mesenchymal stem cells from bone marrow [16], or autologous adipose stem cells in scarred vocal folds [17, 18], with very promising results. The experimental model in the vast majority of studies is based on creating a trauma on the vocal fold and within seven days maximum, there is implantation of stem cells. Our protocol was based on creating a trauma on the vocal folds of an experimental model (rabbit) and adipose-derived stem cells (ADSC) were infused on the scar after 18 months. We compared our results with a control group which did not receive any treatment and a group of experimental models in which we infused hyaluronic acid in the scarred vocal fold (Scheme 1).

## 2. Materials and Methods

### 2.1. Experimental Model

The principle study design was used by several investigators. Our experimental models were three-month-old white male New Zealand rabbits with a body weight between 2.900 Kg and 3.950 Kg. Rabbits were chosen for their relatively small size and low cost as an experimental model but large enough vocal folds to work with. The vocal folds of rabbits also present a three-layered structure like in human beings. All the animal care and experimental procedures were conducted in accordance with the guide of animal experiments and the experimental protocol had been approved by the Scientific Committee of Experimental-Research Center ELPEN and by Veterinary Authority of East Attica Prefecture (pd 160/1991, EU Directive 609/1986).

We used 74 white New Zealand rabbits for the protocol. Sixteen of them were used as control group (normal/control group) to compare our results to normal, uninjured vocal folds. These rabbits were sacrificed by an overdose of phenobarbital sodium intravenously.

### 2.2. Vocal Fold Scarring

**Figure 1:**
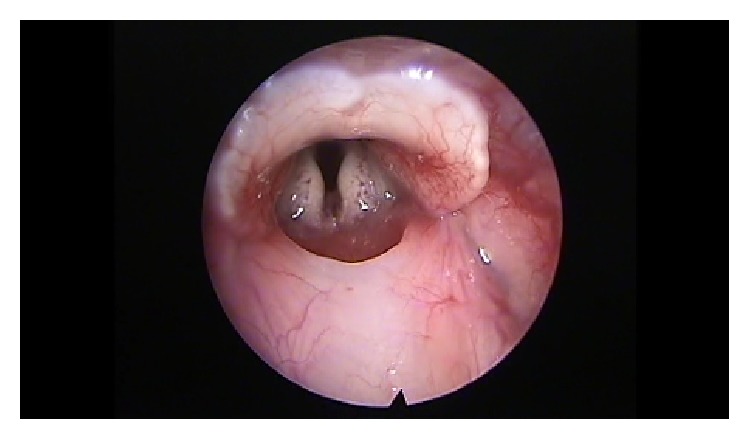
Normal vocal fold.

**Figure 2 fig2:**
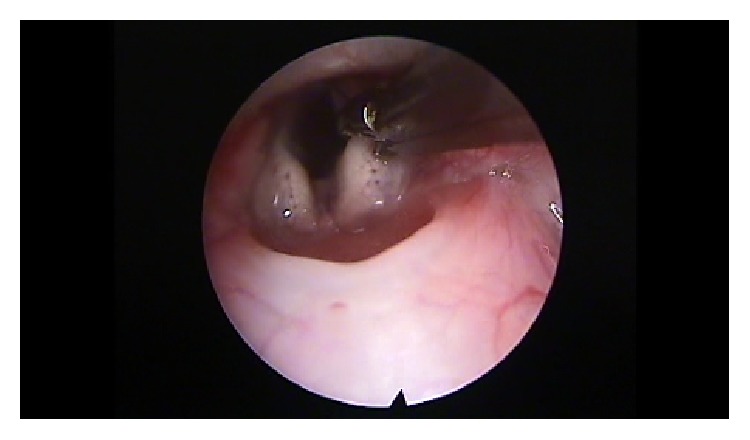
Creation of a trauma using cold instruments.

**Figure 3:**
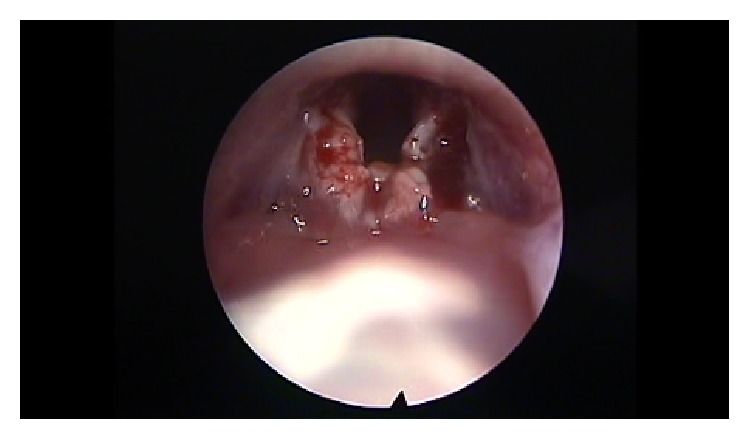
Bilateral trauma on vocal folds.

**Figure 4:**
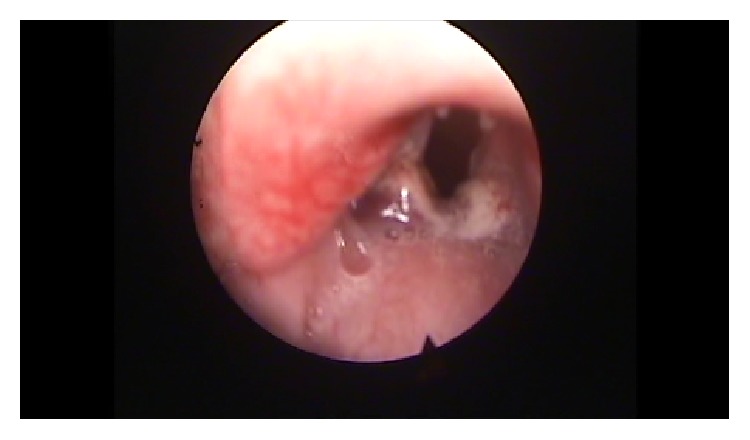
Scar tissue on bilateral vocal folds.

After premedication with glycopyrrolate (0.1 mg/Kg intramuscular) and diazepam (2 mg/Kg intramuscular), all animals were anesthetized with a solution of xylazine (Rompun, 35 mg/kg intramuscular) and ketamine (5 mg/kg intramuscular). The remaining 58 animals were put on a custom-made ramp and with the use of video monitor and a rigid endoscope (4 mm diameter, 0 degrees, MEGA) we proceeded with direct laryngoscopy and visualization of the vocal folds. If any anatomical anomalies were revealed, the animal was ruled out. The scarring procedure was performed with a Hartmann ear forceps, excising epithelium and the lamina propria of the anterior and middle portion of the vocal fold, creating a large defect (Figures 1 [Fig fig2]–3). We administrated immediately postoperatively dexamethasone (Dexamethasone 0.6 mg/Kg) and cefuroxime (Zetagal 30 mg/Kg intramuscularly per day for 3 days, divided into two doses). Seven animals died immediately postoperatively, and 13 died within the following twenty days postoperatively. The remaining 38 animals were transferred to a warrant for about 18 months. After 18 months we performed a direct laryngoscopy to assess the scar and to rule out any experimental models that had granuloma or polyp formation (Figure 4). From the remaining population, we sacrificed 8 rabbits using an overdose of phenobarbital sodium and used them as control group (control/scarred vocal group).

### 2.3. Adipose Mesenchymal Stem Cell Preparation and Characterization

After 18 months 15 rabbits received anesthesia with 50 mg/Kg of ketamine and local anesthesia (lidocaine) in the right inguinal region. We isolated adipose tissue (max 0.2 grams). The incision was sutured in layers.

To isolate ADSC, we digested adipose tissue at 37°C for 40 min using 0, 2% collagenase type I, and 2% bovine serum albumin and neutralized the enzyme activity with Dulbecco's modified Eagle's medium (DMEM) containing 10% fetal bovine serum (FBS). Then, it was centrifuged at 430 ×g (1400) for 10 min without brakes and we resuspended pellet in 100 *μ*L of PBS (buffer), added 10 *μ*L of FcR Blocking Reagent and 10 *μ*L of CD271-APC (10^7^ cells), mixed them well and incubated them for 10 min in the refrigerator (2–8°C), washed cell by adding 500 *μ*L/1 mL PBS (per 10^7^ cells) and centrifuge at 300 ×g for 10 min, aspirated supernatant completely and resuspended cell pellet in 100 *μ*L of PBS (per 10^7^ cells), added 10 *μ*L of FcR Blocking Reagent and 20 *μ*L of Anti-APC MicroBeads per 10^7^ cells, incubated them for 15 min in the refrigerator (2–8°C), washed cell by adding 500 *μ*L/1 mL PBS (per 10^7^ cells) and centrifuge at 300 ×g for 10 min, and aspirated supernatant completely and resuspended pellet in 500 *μ*L of PBS per 10^7^ cells.

### 2.4. Magnetic Separation with LS Columns and Flow Cytometry Analysis

We chose the 25 LS MACS separator columns in order to get 10^8^ cells as the maximum number of labeled cells and 2 × 10^9^ maximum number separators of total cells. The column was placed in the magnetic field and was prepared by rinsing through 2 × 3 mL of PBS each column. Then the cell suspension was applied onto the column. We collected the unlabeled cells that passed through and the column was washed with 3 × 3 mL of buffer. The total effluent was collected (this was the unlabeled cell fraction). We repeated the washing step by adding buffer three times. After the washing step was finished we removed the column and placed it on a 25 mL Falcon tube. A 5 mL buffer was pipetted into the column and immediately we flushed out the magnetically labeled cells by firmly pushing the plunger into the column.

**Figure 5:**
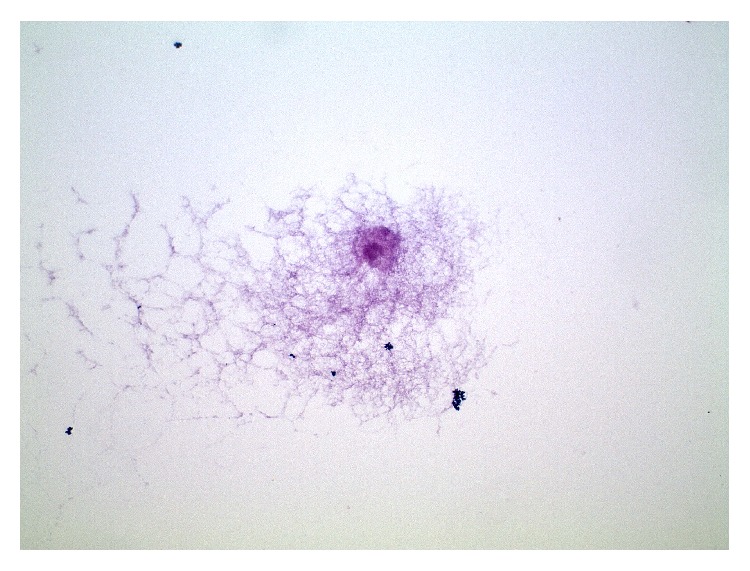
Hematoxylin-eosin. 40x vocal fold: adipose mesenchymal stem cells after isolation, before infusion in animal model.

The total volume of 5 mL ADSC (Figure 5) was collected in the 25 mL Falcon tube and was placed in ice before the infusion in our animal models.

A Cytomics FC500 by Beckman Coulter was used with CXP software for the Cytomics FC500 flow cytometry system version 2.2. We used 500 *μ*L from the total volume of 5 mL ADSC in order to count the total number of cells and their viability, by adding 10 *μ*L of 7-AAD for viable cells, using flow cytometry. A total of 100,000 cells/mL were isolated. Sample analysis was completed typically within 10 minutes.

### 2.5. Vocal Fold Injections

**Figure 6:**
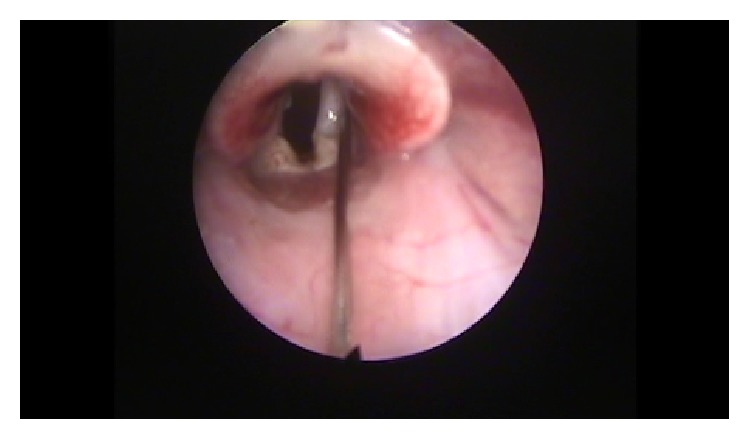
Injection of ADSC.

After three hours (time needed for the isolation of the adipose stem cell), we proceeded with the premedication and anesthesia of all rabbits, according to the previous protocol. All animals were put in a custom-made ramp and with the use of video monitor and a rigid endoscope (4 mm diameter, 0 degrees, MEGA) we proceeded with direct laryngoscopy and visualization of the vocal folds. If any anatomical anomalies were revealed, the animal was ruled out. In the 15 rabbits from which we isolated the adipose stem cells, we proceeded with the injection in both vocal folds of 0.1 mL of solution of stem cells, approximately 10,000 stem cells (Figure 6). The injection was performed in the middle third of the vocal fold, in 0.1 cm of depth. We used a long needle of 27 G, with a marker of 0.1 cm at the end. We took great care to ensure that each injection was an autologous graft (group stem cells). In the remaining 15 rabbits we injected in both vocal folds (group hyaluronan) 0.1 mL of hyaluronic acid (stabilized, non-animal origin hyaluronic acid 25 mg/mL, Gallop Biological Products, China). We administrated immediately postoperatively dexamethasone (Dexamethasone 0.6 mg/Kg) and cefuroxime (Zetagal 30 mg/Kg intramuscularly per day for 3 days, divided into two doses). We lost six rabbits immediately postoperatively.

### 2.6. Dissection

**Figure 7:**
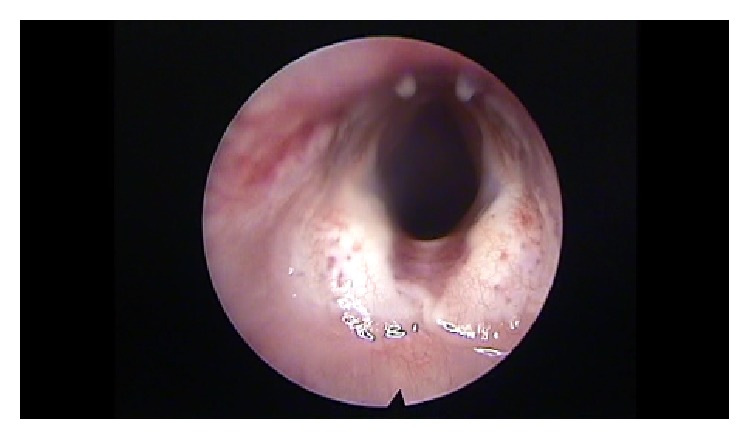
Vocal folds 3 months after the injection of ADSC.

After three months we proceeded with direct laryngoscopy to assess the morphological changes in the vocal folds (Figure 7). The rabbits were sacrificed with an overdose of phenobarbital sodium intravenously. Each larynx was dissected from the thyroid cartilage to the third tracheal ring and put in a solution of 10% formaldehyde. The larynges were finally embedded in paraffin wax and cut into 5 *μ*m thick horizontal sections, covering the whole thickness of the vocal folds.

### 2.7. Histological Staining and Measurements

**Figure 8:**
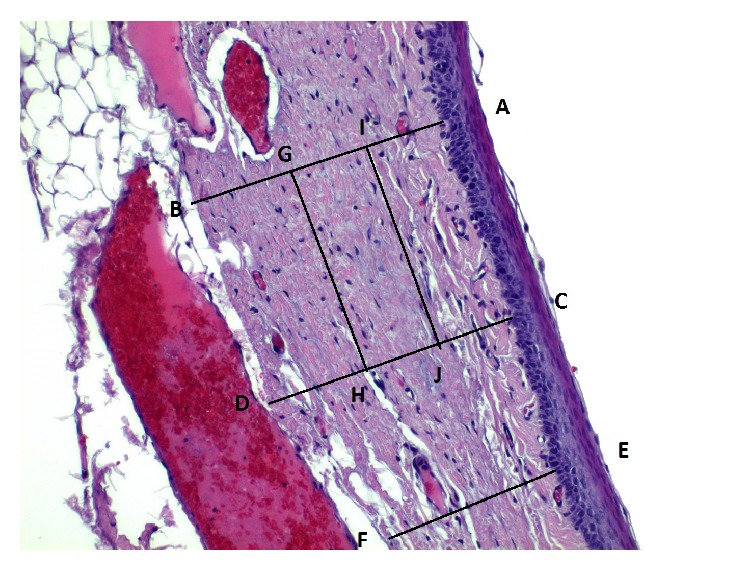
Schematic overview of lamina propria thickness. Lines AB, CD, and EF represent three different points for the calculation of the median. Measurements were performed in mm. Lines GH and IJ represent the division of the lamina propria in three equal-in-thickness layers, SLP, ILP, and DLP.

Staining was made with hematoxylin and eosin and images analysis was made at 10x, 20x, and 40x magnifications (Axiolab ZEISS microscope). Lamina propria thickness was assessed by a hematoxylin and eosin stain at ×10 magnification and was assessed by measuring the distance from the basal lamina of the epithelium down to the thyroarytenoid muscle. Measurements were made at three spots of the lamina propria of each vocal fold, at the middle third of uninjured vocal folds, and at the site of the scar tissue in treated vocal folds. We measured the thickest part, the thinnest part, and an intermediate one and we calculated the median (Figure 8). Collagen deposition through the three layers of lamina propria was assessed with Masson's trichrome staining (Bio-Optica, Milan). Hyaluronic acid is an anionic, nonsulfated glycosaminoglycan that appears in great quantities in the superficial layers of the lamina propria of normal vocal folds but in less quantity in the deep layers. For the assessment we used Alcian blue stain (Sigma Aldrich), which stains glycosaminoglycans including hyaluronic acid into a bluish-green color. Elastic fibers are found in abundance mainly in the intermediate layer of the lamina propria in humans. We used reticulin stain (Reticulin and Orcein Kit, Dako, Denmark) to assess the elastic fibers. These assessments were performed in a double blind way by two examiners, in which the examiners were not informed of the group to which each slide belonged and recorded the results on a subjective scale of 1–3, ranging from mild to important. The elastin was also studied as to the infrastructure of the fibers and graded on a subjective scale of 1–3, ranging from mild to important disorganization and fragmentation of the fibers.

### 2.8. Statistical Analysis

For statistical analysis of the data we used SPSS, version 20.0. The thickness of lamina propria between the different groups was tested by a 1-way analysis of variance (ANOVA). For the structure of the elastin and the density of collagen, hyaluronan, and elastin, we proceeded to logarithmic transformations considering that the variables are not normally distributed (Kolmogorov-Smirnov control, *p* < 0.001). Then we performed comparisons between the groups using the Bonferroni method. Results are depicted in Figures 13–23, using boxplots. The median of results (density) is emphasized by the bold line. The numbers depicted are outlier values, observations in a data set which are far removed in value from the others in the data set. They are unusually large or have an unusually small value compared to the others.

## 3. Results

### 3.1. Laryngeal Morphological Changes

In the untreated group (control B), the surface of 100% vocal folds was irregular, with a scar and stiffness. In the group that received ADSC implantation, the surface of the vocal fold tends to be smoother, with fewer irregularities (Figure 7). In the group that received hyaluronan injection the surface of the vocal fold remains irregular and stiff.

### 3.2. Laryngeal Pathological Changes

#### 3.2.1. Thickness of Lamina Propria

**Figure 9:**
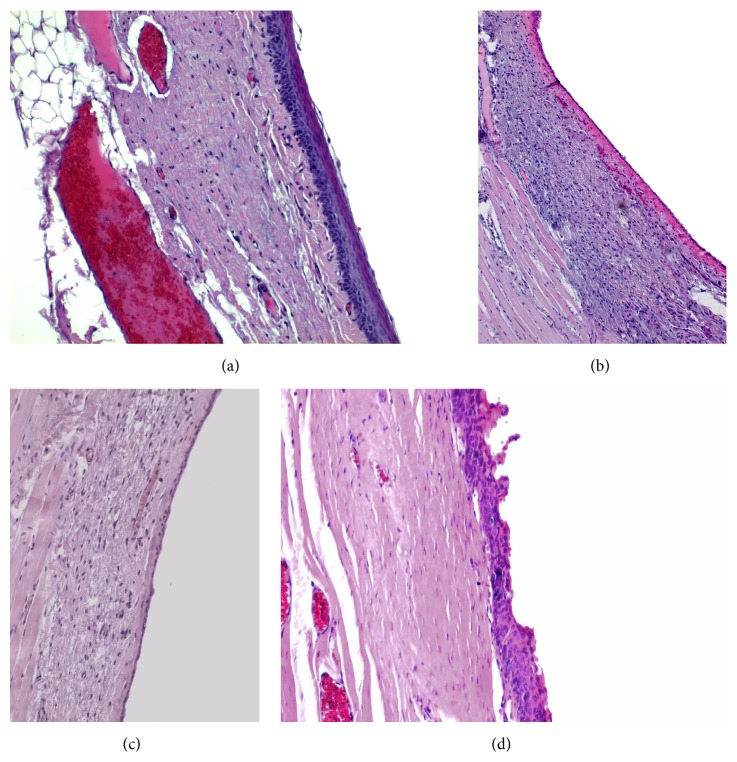
(a) Normal vocal fold. (b) Scarred vocal fold. (c) Injection of ADSC. (d) Injection of hyaluronan.

In the normal population the mean value of the lamina propria is 0.1 mm (maximum: 0.35 mm, minimum: 0.03 mm, Figure 9(a)). In the control group, we can observe that, 18 months after the creation of the lesion and the scar tissue formation, the mean thickness of the lamina propria is considerably increased, mean value of 0.3 mm (maximum: 0.55 mm, minimum: 0.09 mm, Figure 9(b)) (*p* < 0.001). In the group in which stem cells were infused, the thickness of the lamina propria returns to normal values and tends to be of the same values as in the normal group, mean of 0.1 mm (*p* < 0.05, comparing with control group) (maximum: 0.45 mm, minimum: 0.03 mm, Figure 9(c)). In the group in which we injected hyaluronic acid, we can observe that the thickness of the lamina propria is also reduced and tends to return to normal (Figure 9(d)). There is no statistically significant difference between the thickness of the lamina propria in the stem cell group and that in the hyaluronan group.

#### 3.2.2. Collagen

**Figure 10:**
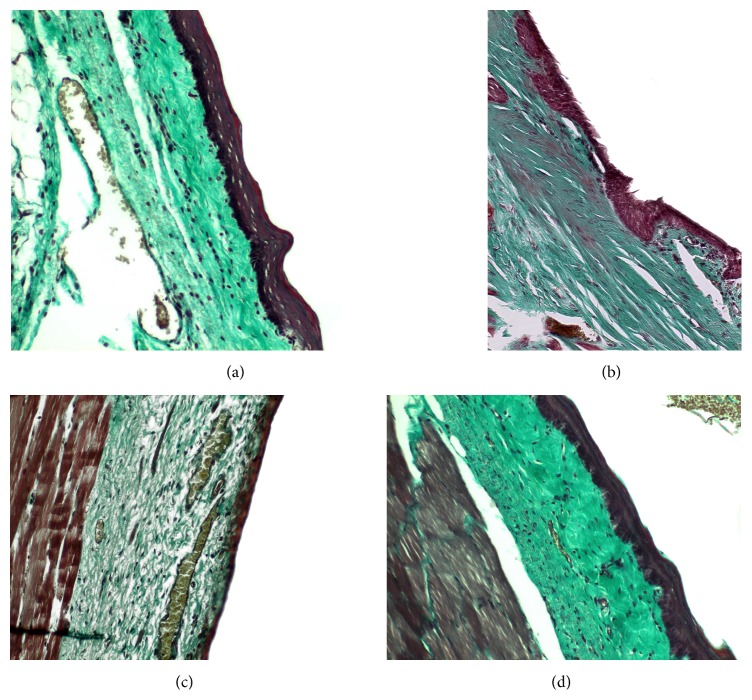
(a) Normal vocal fold. (b) Scarred vocal fold. (c) Injection of ADSC. (d) Injection of hyaluronan.

The collagen in the normal vocal folds is mainly distributed in the intermediate and the deep layers of the lamina propria (Figure 10(a)). In the control group in which we created a scar, we can observe that the amount of collagen is significantly increased in the superficial layers of the lamina propria (*p* < 0.05) when compared to the normal vocal folds (Figure 10(b)). In the intermediate and the deep layers we can see an increase of the density but it is not statistically significant. In the group in which we injected adipose stem cell we can observe that we have a significant reduction of the amount of collagen into the three layers of the lamina propria comparing to the control (superficial *p* value = 0.003, intermediate *p* value = 0.002, and deep *p* value = 0.02, Figure 10(c)). In fact, the collagen levels seem to be identical between the normal group and the stem cell group, in the superficial and the deep layers, and only in the intermediate layers in the stem cell group are reduced (*p* value = 0.009). When compared to the group in which we infused hyaluronan, it seems that the amount of collagen remains increased as it was in the control group (Figures 14 [Fig fig15]–16 and 10(d)).

#### 3.2.3. Elastin

**Figure 11:**
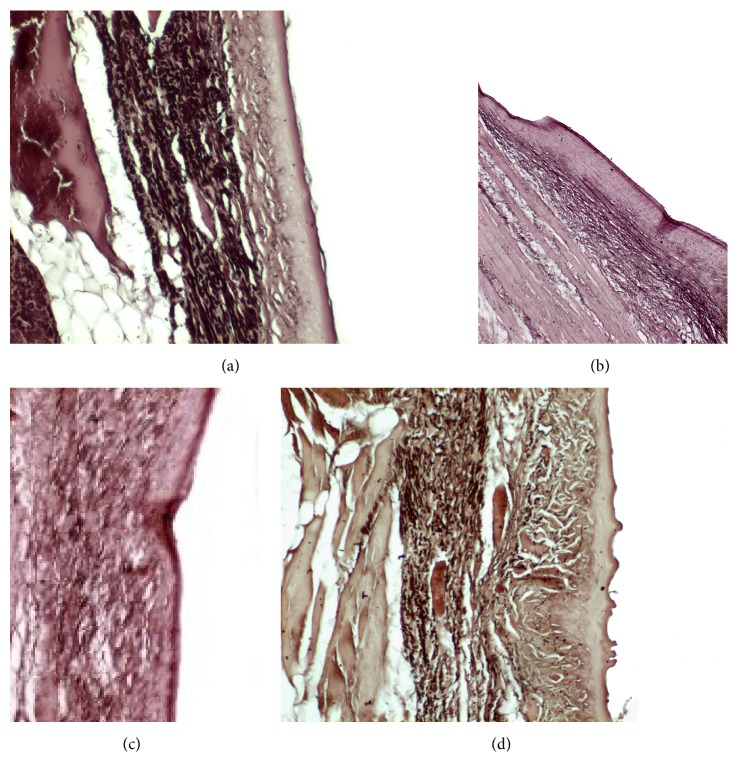
(a) Normal vocal fold. (b) Scarred vocal fold. (c) Injection of ADSC. (d) Injection of hyaluronan.

The elastin is located mostly in the intermediate layer of the lamina propria as seen by the median (Figure 11(a)). In the control group, we observed that when we had formation of a scar tissue, the amount of elastin was reduced in the superficial (*p* value < 0.001) and in the intermediate (*p* value = 0.012) layer, when compared to the normal vocal fold (Figure 11(b)). In the group in which we infused stem cells, although there is an increase of elastin through the three layers of the lamina propria, this difference is not statistically significant (Figure 11(c)). The same is also true for the group in which we injected hyaluronan (Figure 11(d)). There is a tendency to increase in the elastic fibers when compared to the control group but it is not statistically significant. We also observed the structure of the elastin. In the group of normal vocal folds, the structure of the elastin is linear, not disturbed in bundles. In the control group we observed that the elastic fibers were more disorganized, fragmented, and shorter (*p* < 0.05). In the group in which we infused stem cells the structure of elastic fibers returns to normal. In the group in which we infused hyaluronic acid, the elastic fibers also tended to have a more normal structure, as in the normal group and the stem cell group. There is no statistically significant difference between the normal group, stem cell group, and hyaluronan group, although when we take a look at the means and the median between the stem cell group and the hyaluronan group, in the stem cell group elastic fibers tend to be less disorganized (Figures 17 [Fig fig18] [Fig fig19]–20).

#### 3.2.4. Hyaluronic Acid

**Figure 12:**
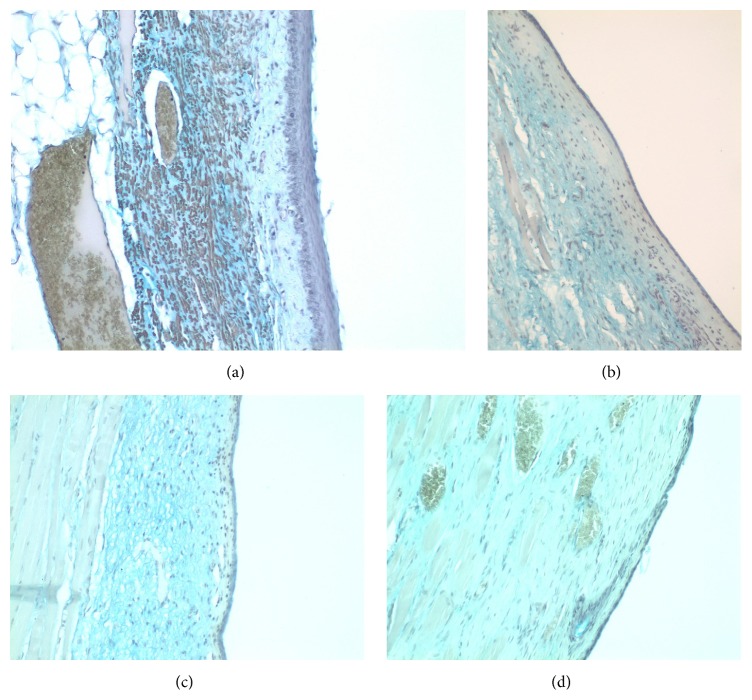
(a) Normal vocal fold. (b) Scarred vocal fold. (c) Injection of ADSC. (d) Injection of hyaluronan.

**Figure 13:**
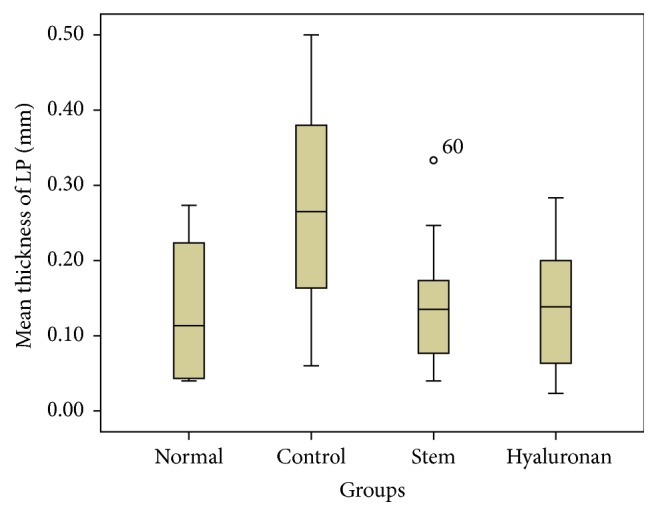
Mean thickness (mm) of the lamina propria (LP), through the different treatment groups. Normal = normal/control group, control = scarred/control group, stem = stem cell group, and hyaluronan = hyaluronan group.

**Figure 14:**
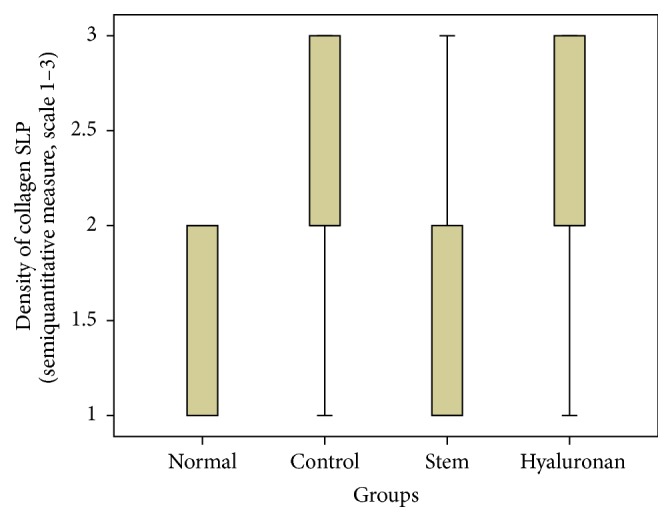
Density of collagen in the superficial lamina propria (SLP). Normal = normal/control group, control = scarred/control group, stem = stem cell group, and hyaluronan = hyaluronan group.

**Figure 15 fig15:**
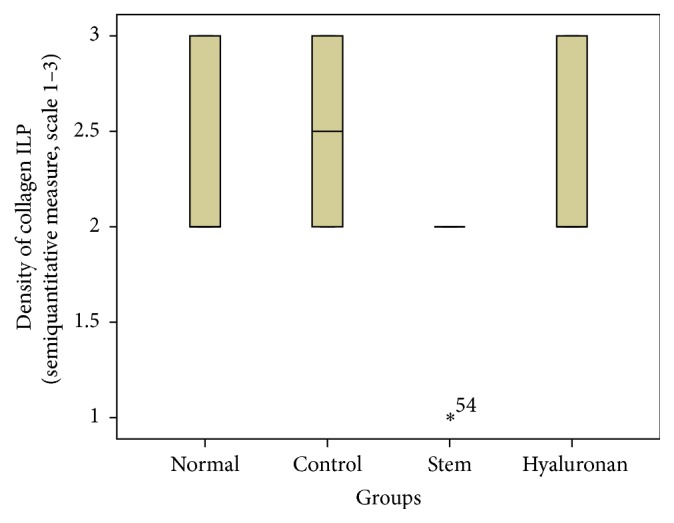
Density of collagen in the intermediate lamina propria (ILP). Normal = normal/control group, control = scarred/control group, stem = stem cell group, and hyaluronan = hyaluronan group.

**Figure 16:**
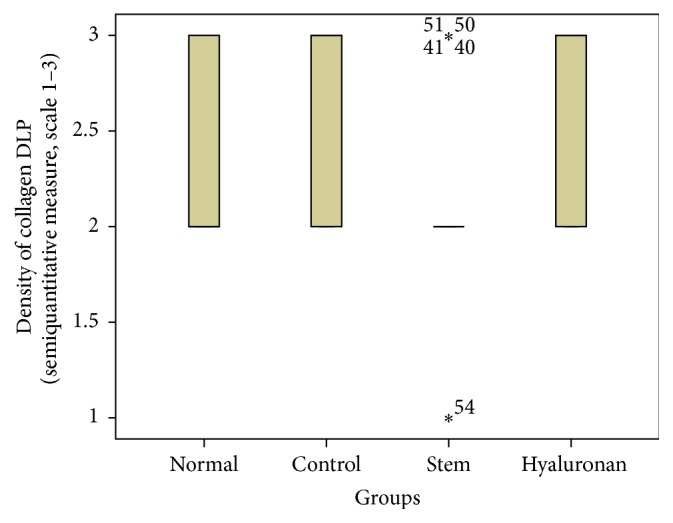
Density of collagen in the deep lamina propria (DLP). Normal = normal/control group, control = scarred/control group, stem = stem cell group, and hyaluronan = hyaluronan group.

**Figure 17:**
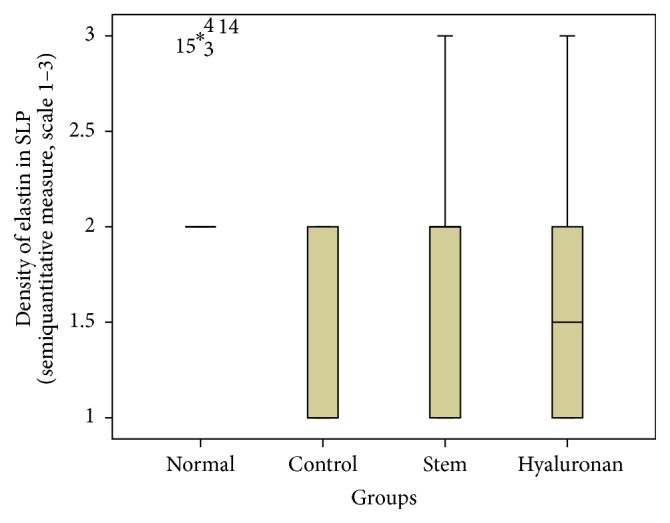
Density of elastin in the superficial lamina propria (SLP). Normal = normal/control group, control = scarred/control group, stem = stem cell group, and hyaluronan = hyaluronan group.

**Figure 18 fig18:**
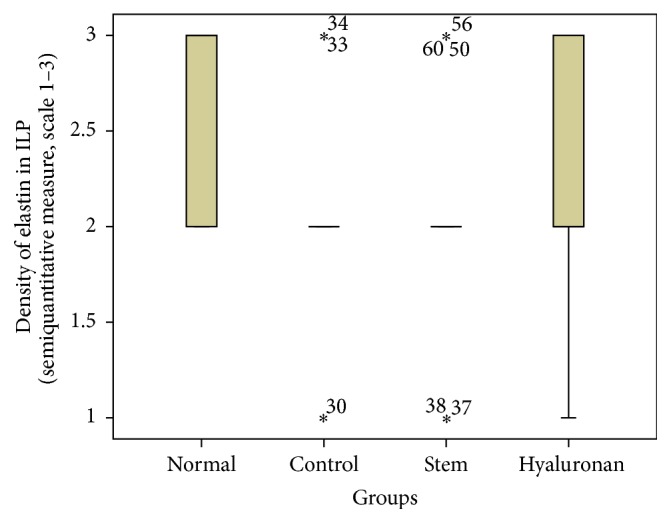
Density of elastin in the intermediate lamina propria (ILP). Normal = normal/control group, control = scarred/control group, stem = stem cell group, and hyaluronan = hyaluronan group.

**Figure 19 fig19:**
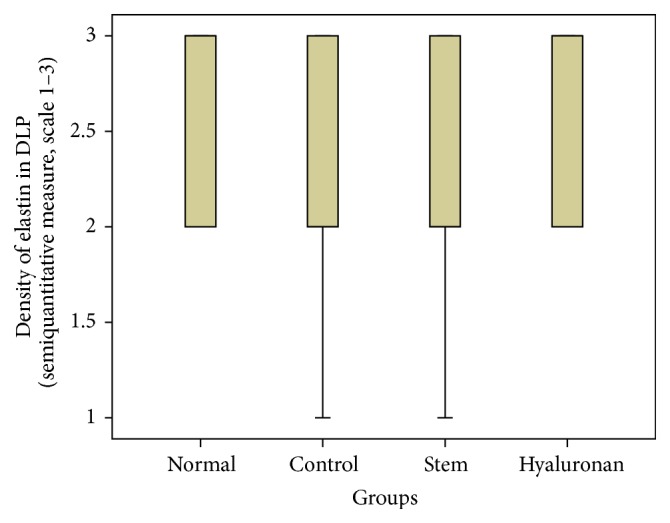
Density of elastin in the deep lamina propria (DLP). Normal = normal/control group, control = scarred/control group, stem = stem cell group, and hyaluronan = hyaluronan group.

**Figure 20:**
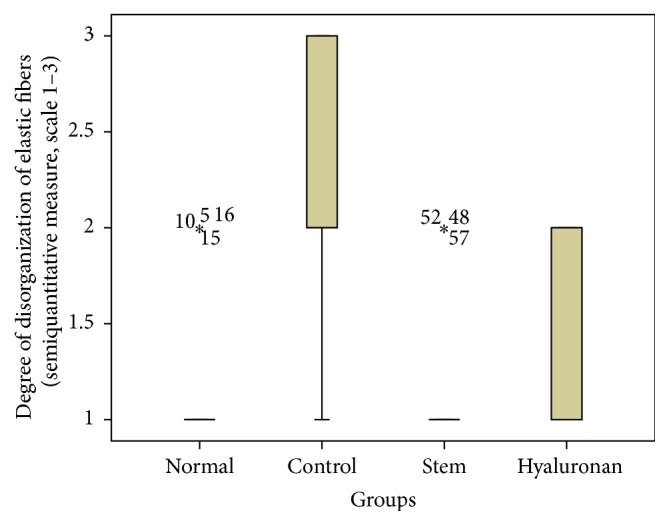
Level of disorganization of elastic fibers. Normal = normal/control group, control = scarred/control group, stem = stem cell group, and hyaluronan = hyaluronan group.

**Figure 21:**
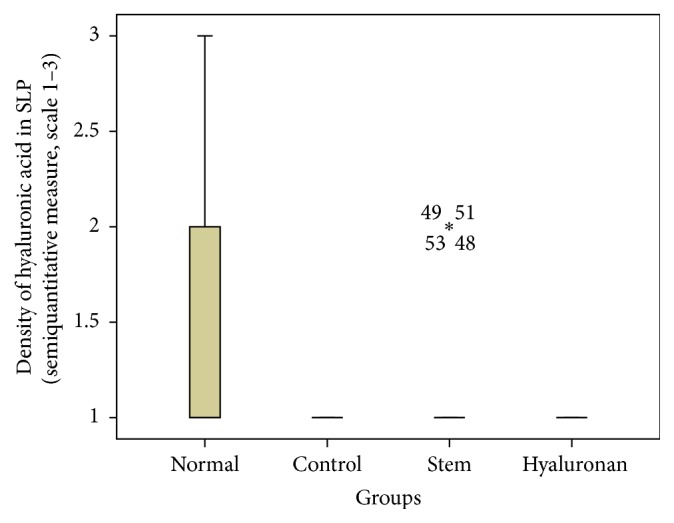
Density of hyaluronic acid in the superficial lamina propria (SLP). Normal = normal/control group, control = scarred/control group, stem = stem cell group, and hyaluronan = hyaluronan group.

**Figure 22 fig22:**
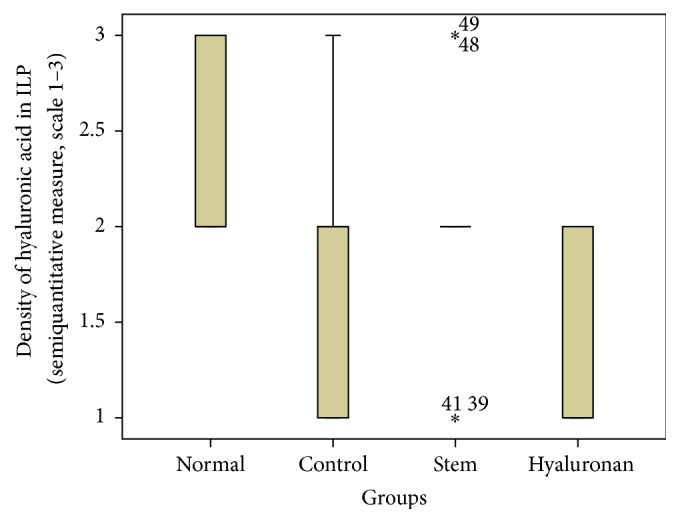
Density of hyaluronic acid in the intermediate lamina propria (ILP). Normal = normal/control group, control = scarred/control group, stem = stem cell group, and hyaluronan = hyaluronan group.

**Figure 23:**
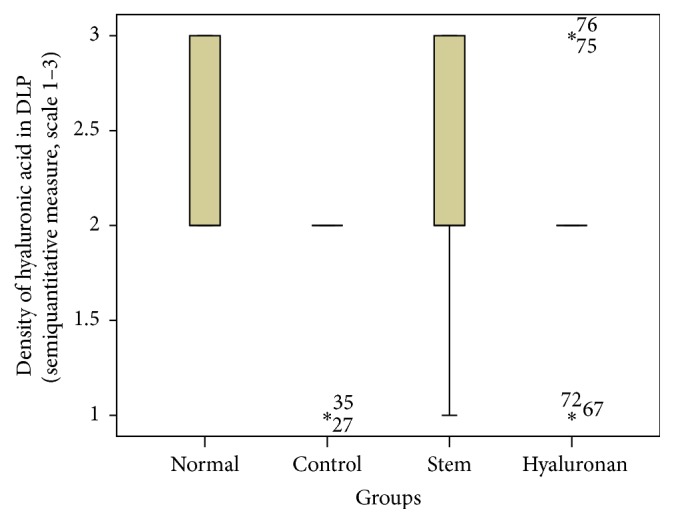
Density of hyaluronic acid in the deep lamina propria (DLP). Normal = normal/control group, control = scarred/control group, stem = stem cell group, and hyaluronan = hyaluronan group.

In the normal vocal folds the hyaluronic acid is also mainly distributed in the intermediate and the deep layers of the lamina propria (Figure 12(a)). In the control group, in the presence of the scar tissue we observed that we have a statistically significant reduction of the amount of hyaluronan in the superficial layers (*p* = 0.017) and the intermediate layers (*p* = 0.007), when compared to the normal vocal folds (Figure 12(b)). When we compare the infused adipose stem cells group to the control group there is an increase of the amount of hyaluronan through the three layers of the lamina propria which is not statistically significant (Figure 12(c)). Also, when we compare the amounts of hyaluronan between the normal group and the stem cell group there is no statistically significant difference either. In the group where we infused hyaluronan into the scarred vocal folds, the amount of hyaluronan in the vocal folds remains reduced, as it was in the scarred vocal folds, and in the intermediate layer was even more reduced (*p* = 0.02, Figure 12(d)). When compared to the normal vocal folds, the amount of hyaluronan is reduced in the superficial and in the intermediate layer of the lamina propria (Figures 21 [Fig fig22]–23).

## 4. Discussion

Vocal folds have a highly specific structure, based on a specific distribution of the different cellular population and the different constituents of the extracellular matrix. When there is a trauma on the vocal fold, a high intensity inflammatory process takes place, in order to restore the structure and the function of the vocal fold. In case of a great trauma and as a consequence an intense inflammatory response, the healing process can lead to the formation of a scar tissue. This scar tissue mostly located in the lamina propria of the vocal fold adds great stiffness and interferes with the normal vibratory properties of the vocal fold by losing the three-layered structure of the lamina propria. Clinically patients present with dysphonia, hoarseness, loss of vocal range, and strain. Numerous treatment modalities and injection materials have been applied in order to treat the glottal insufficiency caused by scar tissue or vocal paralysis. None of them led to an optimal result.

According to Arnold [19], the requirements for injection materials include minimal tissue response, absence of oncogenicity, ease of injection, nonabsorbability, and absence of migration. Injection of autologous fat has been used for the last 30 years. The great disadvantage of this method is that the adipose tissue used must be carefully rinsed with saline to remove any clots. Then it must be harvested in very small pieces and injected using a special Bruening Pressure Syringe, due to its elevated viscosity. Over the years, surgeons observed that the injected fat can be absorbed or migrate during the months following the intervention [20]. Hyaluronic acid-based injection materials have viscoelastic properties that resemble those of human vocal folds and present a good safety profile [21]. The major disadvantage is that the voice improvement generally lasts about 4–6 months, due to the degradation of hyaluronic acid.

Over the last years, much attention has been directed in the cell therapy research and regenerative medicine. Regenerative cell therapy involves the delivery of autologous or nonautologous mesenchymal stem cell, with the purpose of tissue and organ regeneration and reconstruction. Level of evidence was progressive within the years. Hanson et al. [22] proved that human vocal fold fibroblasts have the same immunophenotypic characteristics and cell surface markers as bone marrow and adipose tissue mesenchymal stem cells. In another study, human adipose stem cells were isolated and cultured in a fibrin hydrogel with a growth factor supplementation and compared to cadaveric human vocal folds [23].

This study gives a great amount of information concerning the pathological changes following the implantation of adipose stem cell in a chronic vocal fold wound. In our study the density of collagen after the implantation of adipose stem cell is reduced significantly at the same levels as the normal, uninjured vocal folds. In studies where adipose stem cells have been used, Liang et al. [24] and Hu et al. [25] had the same results in an acute model of scarred vocal fold. Hong et al. [18] had also a reduction of collagen levels, although the adipose stem cells were not autologous. On the contrary Cedervall et al. [15] failed to prove difference concerning collagen levels between scarred vocal folds and treated ones, where the cell therapy was based on human embryonic stem cells in an acute model of scarred vocal folds.

When we want to compare the effects on quasi-mature scar regardless of the type of mesenchymal stem injected, we see that Svensson et al. [26], in a study where they injected human mesenchymal stem cells in a mature scar, did not observe any statistically significant difference between scarred and treated vocal folds. Thibeault et al. [27], while injecting autologous fibroblasts in a mature scar having been cultured or not in an extracellular matrix environment, observed that the density of collagen was even greater in the treated than in the normal vocal folds. This can be explained by the fact that mature fibroblasts probably do not have the potential to modulate the scar and that their implantation just as any additionally surgical intervention reboots the inflammatory process of healing.

In our study we failed to prove an increase of elastic fibers in the group of animals that were treated with adipose stem cell, although there is a clear tendency. The fact is that we demonstrate a significantly better structure and rearrangement of the fibers of elastin, when compared to the scarred/untreated group. We observed the same result in the vocal folds which were treated with hyaluronic acid. This result could be explained based on the hyaluronan anti-inflammatory profile as shown by the study of Hanson et al. [28]. The presence of hyaluronan in an inflammatory process (the injection of any material in the vocal fold leads to a minimum of trauma and as a consequence to a minimum inflammatory process) could change the phenotype of macrophages recruited in the spot, promoting the anti-inflammatory phenotype of the macrophages in the detriment of the proinflammatory phenotype. Svensson et al. [16], in their study, where they injected human mesenchymal stem cells in an acute model of scarred vocal folds, did not observe any statistically significant difference.

There is no great amount of literature on the effects of cell therapy on the hyaluronan level in a scar tissue of the vocal folds. In our study, the levels of hyaluronic acid were significantly reduced in a chronic scar of vocal folds and returned to the same levels as in normal vocal folds. When we want to compare the studies in function of injection of ADSC, regardless of the maturity of the scar tissue, Hu et al. [25] had the same results in an acute model of vocal fold scar. In a study where mouse bone marrow-derived clonal mesenchymal stem cells were injected in an acute vocal fold wound in a rabbit model, the hyaluronic acid levels were improved when compared to the scarred (untreated) group [29]. In the group which was treated with hyaluronic acid, the levels of hyaluronan remained decreased when compared to normal vocal folds. This can be explained by the fact that hyaluronic acid is an inactive material without any potential of proliferation by itself. Studies have shown that it is absorbed and the filler effect lasts some months.

The major difference between the groups treated with mesenchymal stem cells and hyaluronic acid is the important dissolution of the excess of collagen fibers in the stem cell group through all the three layers of the lamina propria (*p* < 0.05). Hyaluronic acid tends to be increased in the group treated with stem cells, compared with the group treated with hyaluronic acid; this difference was statistically significant in the intermediate lamina propria.

In our study we used the mesenchymal stem cells derived from a minimum amount of autologous fat while in all other studies the mesenchymal cells derived from bone marrow or the placenta, a nonautologous source. Few studies exist which use adipose-derived stem cells but the literature is growing [17, 18, 24–26]. The advantages of our method are easiness, painless preparation, and greater stability. Our method does not require special storage conditions of preparation, until the time of isolation of mesenchymal stem cell, and does not involve ethical issues comparing with embryonic stem cells. It is the only study in which every rabbit received autologous adipose-derived stem cell.

The laryngoscopy was performed with the aid of mouth expander and a rigid endoscope of 0°/4 mm as opposed to the use of pediatric Pillings pediatric endoscope used in the majority of studies. This allows us to have a better exposure of the operative field.

In the past studies the implantation of mesenchymal stem cells had been some days (5–7) after the creation of the vocal cord injury [14, 16, 24, 30, 31] or even before the lesion [32]. Quinchia Johnson et al. [33] went a step further and studied the effects of autologous bone marrow mesenchymal stem cells one month after the trauma of the vocal folds. This means that the implant is in the middle of intense inflammatory reaction. In the study we present, implantation was conducted 18 months after the injury of vocal cord. Past studies have found that the scar tissue is stable after 6 months of the vocal cord injury [34]. Hiwatashi et al. [17] and Svensson et al. [26] studied the effects of mesenchymal stem cells two months after the injury. Thibeault et al. implanted mesenchymal stem cells after three months of scarification [27]. Based on the above, the study we present is the first study with implantation of autologous mesenchymal chronic scar tissue after so long (18 months).

In the present study we created three groups (untreated chronic scar group, adipose stem cells group, and hyaluronan group) and we compared the pathological findings (density of collagen, elastin, and hyaluronan and thickness of the lamina propria) between two treatment modalities and one control group. In previous studies [15, 16], the effects of injection of mesenchymal stem cells were compared to the injection of saline. It is the only study that compares the effects of mesenchymal stem cells on a chronic vocal fold scar to the effects of hyaluronic acid and to an untreated chronic scar.

Finally, on pathological sections differentiated mesenchymal cells from adipose tissue were identified. The identification was performed using 7-AAD.

Although many interesting points have been made in this research, it confirms that the amelioration in the microstructure of the vocal fold coexists with the amelioration of the vibratory properties also. Restoration of a chronic vocal wound with injection of ADSC has to improve the plasticity and rheology of the vocal folds.

## 5. Conclusions

Restoration of a chronic vocal wound still remains a great challenge. The data presented indicate that the transplantation of adipose-derived mesenchymal stem cells in a chronic scar can lead to an anatomical regeneration of the vocal fold by dissolution of the excessive collagen fibers forming the scar tissue and restoration of the normal structure of the elastic fibers. Further studies have to be conducted in order to assess the improvement of the viscoelastic properties.
